# Supplementary material for: Metabolic switch in the aging astrocyte supported via integrative approach comprising network and transcriptome analyses
Source: Aging (Albany NY). 2023 Apr 18;15(19):9896–912. doi: 10.18632/aging.204663 (PMC10599759; doi:10.18632/aging.204663)
Supplement: Supplementary Figures [file aging-15-204663-s002.pdf]

SUPPLEMENTARY FIGURES

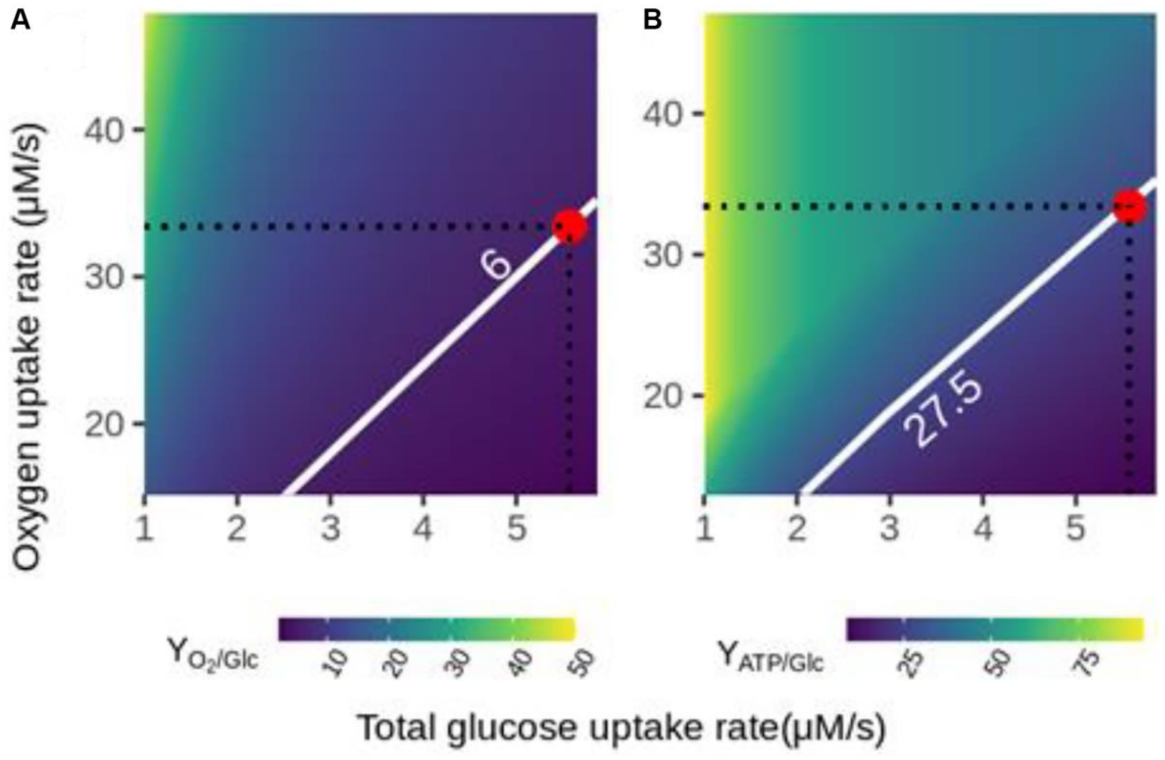

Supplementary Figure 1. Glucose yield is consistent with aerobic metabolism. Phenotypic phase planes are shown as two-dimensional color maps. The Flux Balance Analysis (FBA) solution is represented by the red-filled circle. The white piecewise line depicts the specific contour level of the solution. (**A**) Oxygen molecules spent per molecule of glucose. (**B**) ATP molecules produced per molecule of glucose.

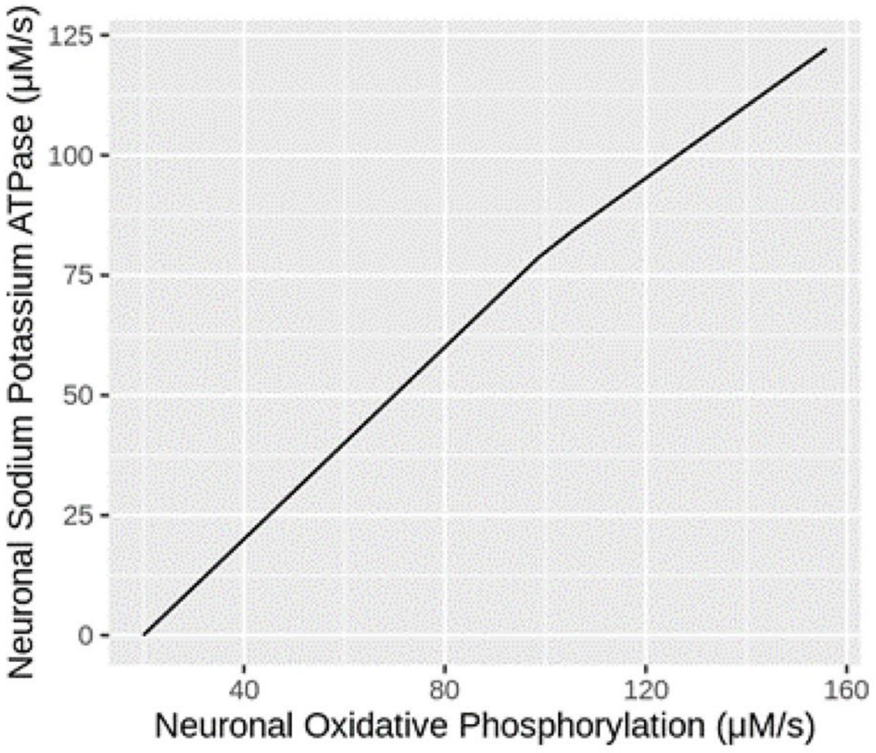

Supplementary Figure 2. Flux coupling between sodium removal and oxidative phosphorylation in neurons.

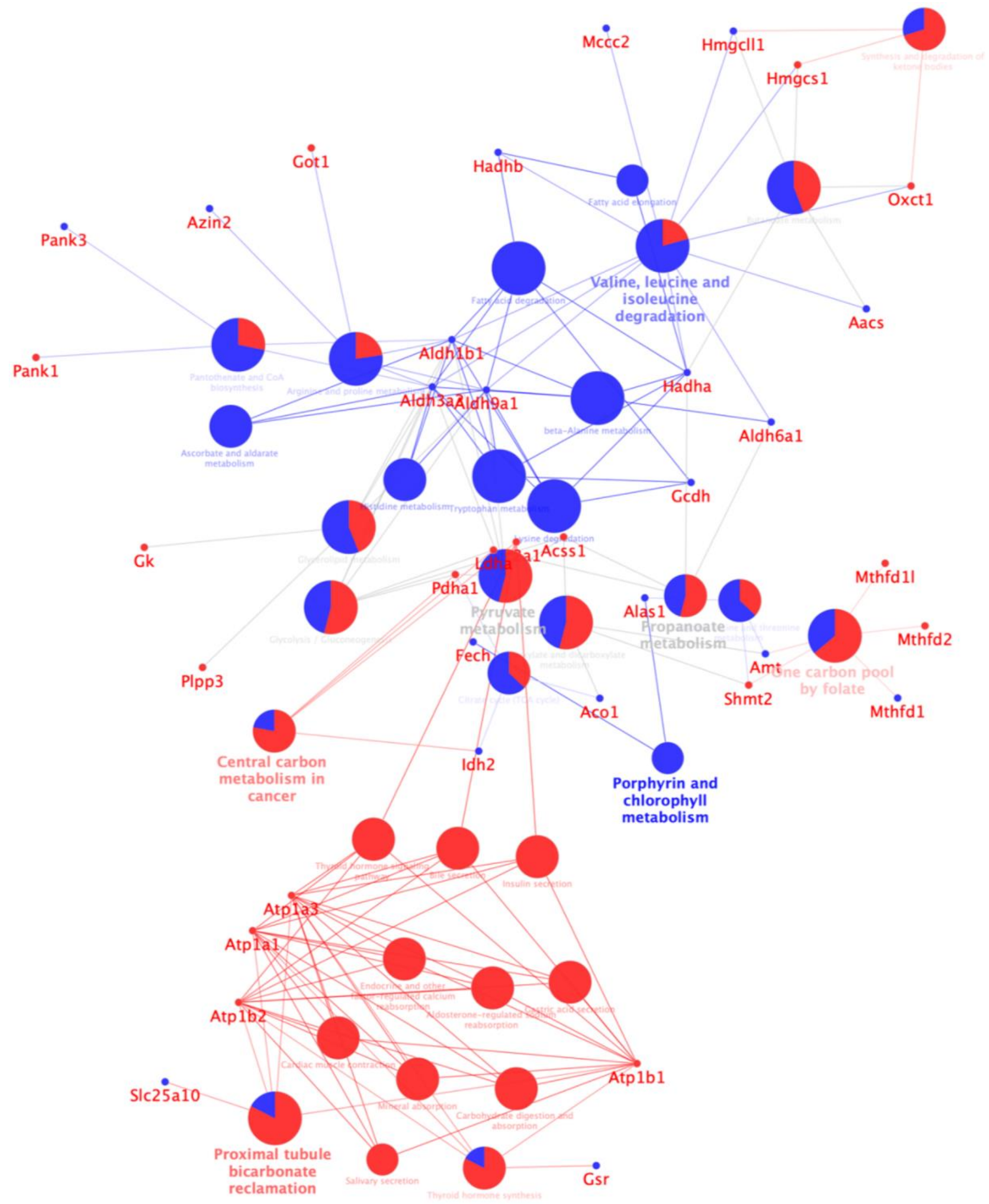

Supplementary Figure 3. Uncurated KEGG enrichment diagram for differential hub genes in the neuron during neurotransmission.

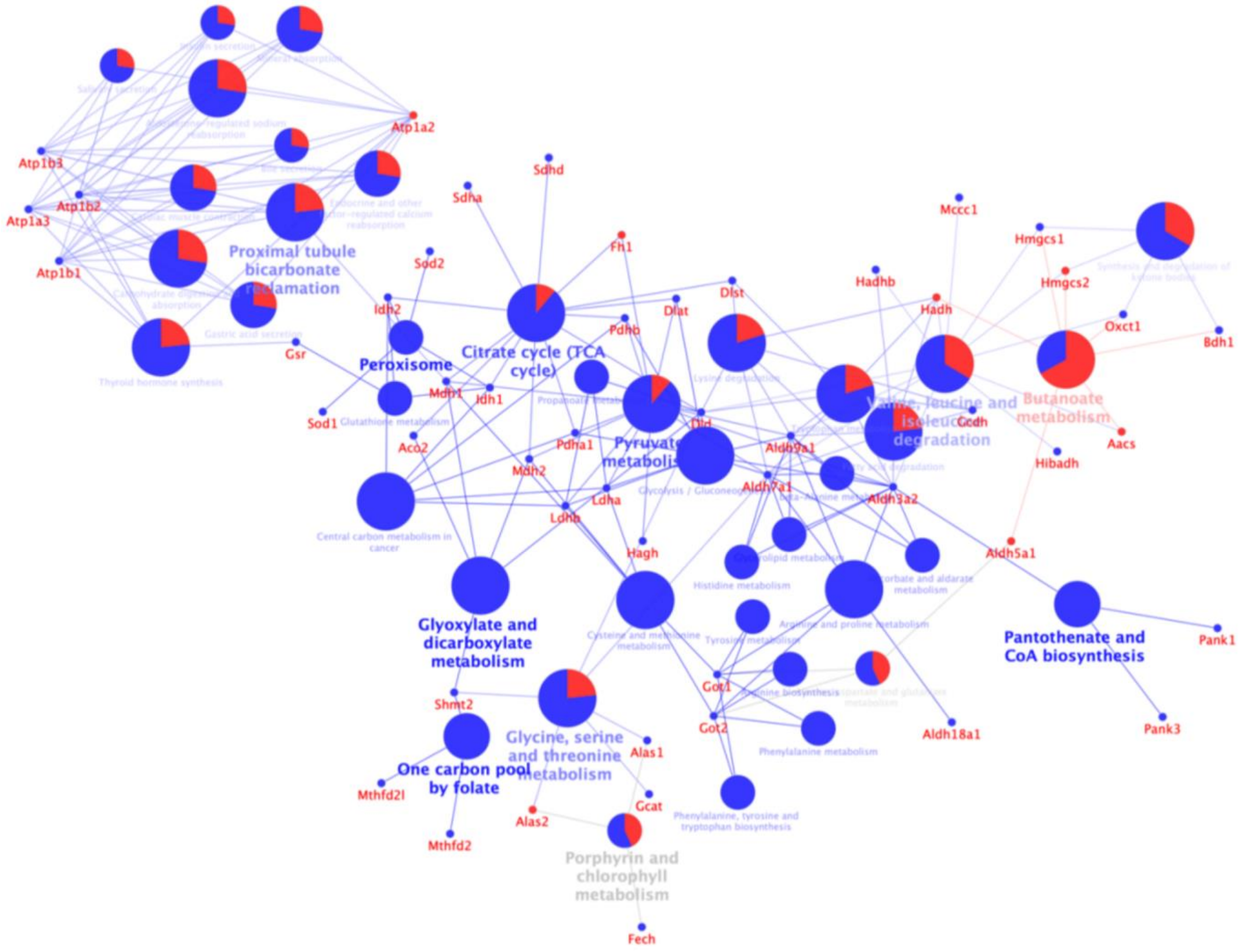

Supplementary Figure 4. Uncurated KEGG enrichment diagram for differential hub genes in the neuron during brain aging.

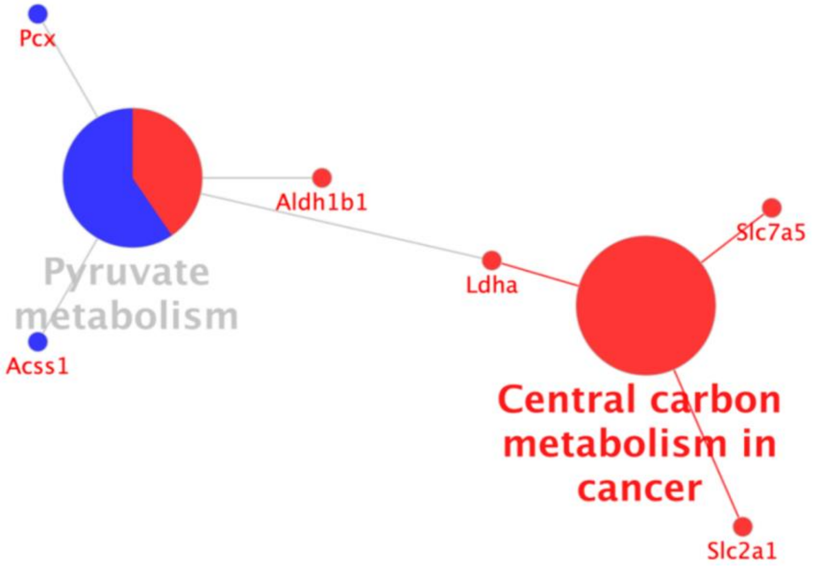

Supplementary Figure 5. Uncurated KEGG enrichment diagram for differential hub genes in the astrocyte during neurotransmission.

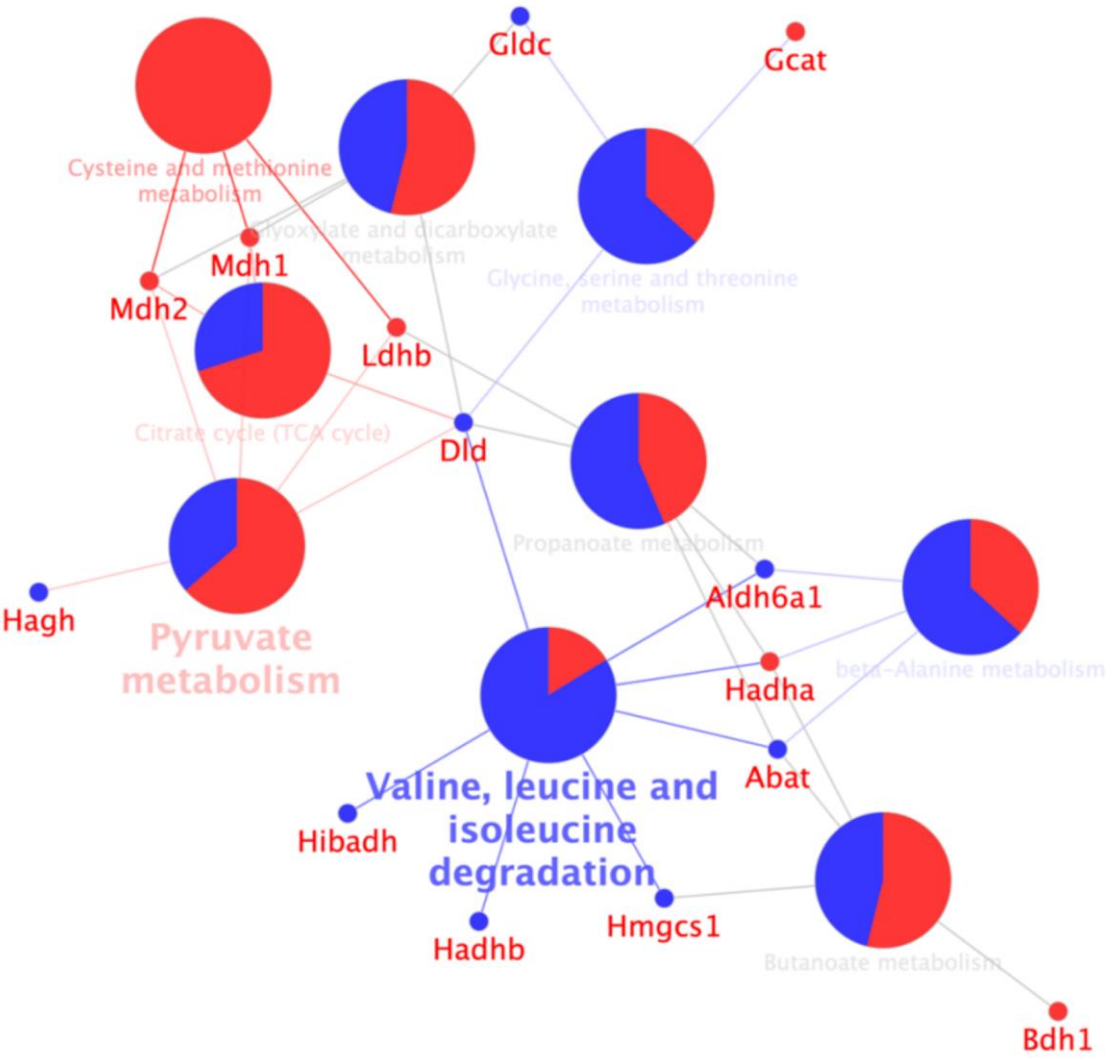

Supplementary Figure 6. Uncurated KEGG enrichment diagram for differential hub genes in the astrocyte during brain aging.
