# Supplementary material for: Metabolic switch in the aging astrocyte supported via integrative approach comprising network and transcriptome analyses
Source: Aging (Albany NY). 2023 Apr 18;15(19):9896–912. doi: 10.18632/aging.204663 (PMC10599759; doi:10.18632/aging.204663)
Supplement: Supplementary Tables 1 and 9 [file aging-15-204663-s003.pdf]

SUPPLEMENTARY TABLES

Supplementary Table 1. Optimal fluxes relevant to the neuron-astrocyte metabolic network during neurotransmission.

	Name	Reaction	Flux	Sensibility
EX_o2(e)	Oxygen uptake	o2[e] <--	−33.425896	0.00000e+00
NaEX_Neuron	Neuronal sodium accumulation rate under stimulation	na1[e] <=> na1[cN]	350.000024	0.00000e+00
NaKt_Neuron	Neuronal sodium-potassium ATPase pump (sodium removal)	atp[cN] + h2o[cN] + 2.0 k[I] + 3.0 na1[cN] --> adp[cN] + h[cN] + 2.0 k[cN] + 3.0 na1[I] + pi[cN]	118.045878	0.00000e+00
L-LACTt2r_Int	L-lactate reversible transport vía proton symport Interstitial and Synapse	h[I] + lact-L[I] <-- h[cA] + lac-L[cA]	−6.912674	0.00000e+00
L-LACTt2r_Neuron	L-lactate reversible transport vía proton symport Neuron	h[I] + lact-L[I] --> h[cN] + lac-L[cN]	6.912674	0.00000e+00
GLUVESSEC_Neuron	L-glutamate secretion vía secretory vesicle (ATP driven) Neuron	atp[cN] + glu-L[cN] + h2o[cN] --> adp[cN] + glu-L[I] + h[cN] + pi[cN]	4.137608	0.00000e+00
GLNtN1_Int	Glutamine transporter Interstitial and Synapse	gln-L[I] + h[cA] + na1[i] <=> gln-L[cA] + h[i] + na1[cA]	−4.137608	0.00000e+00
ATPS4m_Neuron	ATP synthase (four protons per one ATP) Neuron	adp[mN] + 4.0 h[cN] + pi [mN] --> atp[mN] + h2o[mN] + 3.0 h[mN]	155.943088	−2.775558e-16
ATPS4m	ATP synthase (four protons per one ATP) Astrocyte	adp[mA] + 4.0 h[cN] + pi [mA] <-- atp[mA] + h2o[mA] + 3.0 h[mA]	−0.016660	8.63000e+00
GLCt1r	Glucose transport (uniport) Astrocyte	glc-D[e] --> glc-D[cA]	3.452172	6.65200e+01
GLCt1r_Neuron	Glucose transporter Neuron	glc-D[e] --> glc-D[cN]	2.120199	6.00000e+01
ATPtm_Neuron	ADT/ATP transporter, mitochondrial Neuron	adp[cN] + atp[mN] --> adp[mN] + atp[cN]	155.943088	0.00000e+00
PYK_Neuron	Pyruvate kinase Neuron	adp[cN] + h[cN] + pep[cN] --> atp[cN] + pyr[cN]	4.240398	0.00000e+00
PYK	Pyruvate kinase Astrocyte	adp[cA] + h[cA] + pep[cA] --> atp[cA] + pyr[cA]	6.896014	0.00000e+00

The lactate shuttle is active in both directions; L-LACt2r_Int is the efflux from the astrocyte, and L-LACt2r_Neuron corresponds to the influx to neurons. Also, the glutamate-glutamine cycle was active for neuronal glutamate export (GLUVESSEC_Neuron) and glutamine efflux from astrocytes (GLNtN1_INt).

Supplementary Table 9. List of aging-related terms found among the functional annotation of differential hub genes.

Term	Database
GO:0007568~aging	Gene ontology - biological process
GO:0007569~cell aging	Gene ontology - biological process
GO:0010259~multicellular organism aging	Gene ontology - biological process
m_LongevityPathway:The IGF-1 Receptor and Longevity	BioCarta (online maps of metabolic and signaling pathways)
